# The complex stacking disorder of Fe- and Ru-based 1,1′-(3,6-pyrazabolyl)metallocenes

**DOI:** 10.1107/S2052520625009758

**Published:** 2026-02-01

**Authors:** Berthold Stöger, Alexandr Virovets, Mischa Wenisch

**Affiliations:** a X-Ray Centre TU Wien Getreidemarkt 9 1060Vienna Austria; b Institute of Inorganic and Analytical Chemistry Goethe Universitaet Frankfurt Max-von-Laue-Str. 7 60438Frankfurt Germany; University of Geneva, Switzerland

**Keywords:** OD theory, diffuse scattering, synchrotron radiation, metallocenes

## Abstract

1,1′-(3,6-Pyrazabolyl)ferrocene [Fc(BHpz)_2_] and the corresponding ruthenocene [Rc(BHpz)_2_] crystallize as order–disorder (OD) structures with layers of *Pma*(*m*) symmetry. Since the *m*_[100]_ reflection and the *a*_[010]_ glide reflection planes of adjacent layers do not overlap, given one layer, the adjacent layer can be placed in four distinct, but geometrically equivalent, positions. There are accordingly four polytypes of a maximum degree of order (MDO). One analyzed Fc(BHpz)_2_ crystal was composed of a mixture of the MDO_1_ polytype in four distinct orientations and the MDO_3_ polytype. A second crystal was essentially a single crystal of one out of two orientation of MDO_3_. In two analyzed crystals of Rc(BHpz)_2_, the MDO_1_ polytype (*P*1) was the major polytype and was observed in four or two orientation states, respectively. Pronounced diffuse scattering is attributed to a complex disorder, which cannot be explained by simple growth models. One Rc(BHpz)_2_ crystal features additional peaks in the diffraction pattern attributed to fragments of MDO_3_.

## Abstract 2

The ferrocene Fc(BHpz)_2_ and the ruthenocene Rc(BHpz)_2_ belong to the same order–disorder (OD) polytype family and feature a complex stacking disorder, with different ordered and disordered domains in the same crystal.

## 1. Introduction

Development of automated computer-controlled diffractometers and crystallographic software for single crystal X-ray diffraction (SC-XRD) analysis has dramatically changed the attitude of researchers. The method, being initially aimed at determination of the crystal structure of compounds with known chemical formula and obvious atomic connectivity, turned into a powerful chemical analytical tool aimed at determining an *a priori* unclear composition and molecular structure. Not surprisingly, back in the 1990s SC-XRD was already denoted as a ‘first-resort analytical tool’ (Hope & Karlin, 1994 ▸). However, ‘routine’ SC-XRD is based on some assumptions, more or less obvious and therefore more or less commonly forgotten. It is a common belief that it does not matter which single crystal of given polymorphic modification of given chemical compound is taken for the SC-XRD, the resulting crystal structure will be virtually the same, within experimental errors, of course. Recently we experimentally proved that this is not exactly true if one takes into account not only the micro-, but also the macrostructure of the crystal. The presence of such crystal-dependent features such as order–disorder (OD) phenomena (stacking faults in this case) may manifest itself in whole-molecule disorder (Peresypkina *et al.*, 2022 ▸), which varies from crystal to crystal even within the same sample. Some crystals at first sight seemed to be perfectly ordered, while others showed significant disorder, up to 76:24%.

Alongside the frequently underestimated effect on the interpretation of the results of crystal structure determination by SC-XRD, the OD phenomena can in many cases influence physical properties such as microporosity (Meekel *et al.*, 2023 ▸), electronic and phononic band structures (Roth & Goodwin, 2023 ▸) or effects related to correlated disorder in MOFs [mass transport, sorption *etc*. (Cliffe *et al.*, 2014 ▸)].

In this context, we present the pyrazabole-bridged ferrocene 1,1′-(3,6-pyrazabolyl)ferrocene [Fc(BHpz)_2_] and the analogous ruthenocene [Rc(BHpz)_2_] (Scheme 1). Fc(BHpz)_2_ is used as a precursor of a Lewis superacid (Henkelmann *et al.*, 2022 ▸). Crystals of both compounds belong to the same polytype family and feature one-dimensional diffuse scattering owing to stacking disorder. As in many such cases, the ambiguity of the stacking arrangement can be explained by application of the OD theory (Dornberger-Schiff & Grell-Niemann, 1961 ▸). The OD theory is a generalization of classical crystallography, taking into account the limited range of interatomic interactions: members of an OD family of structures are all locally equivalent. Yet, owing to local pseudo-symmetry, they may differ on a longer range. They can be ordered or disordered, but are locally built according to a strict *symmetry principle* (Fichtner, 1979*a* ▸). Here, we will apply the OD formalism to the crystal structures of the title compounds, which feature a surprisingly complex crystallization behavior.
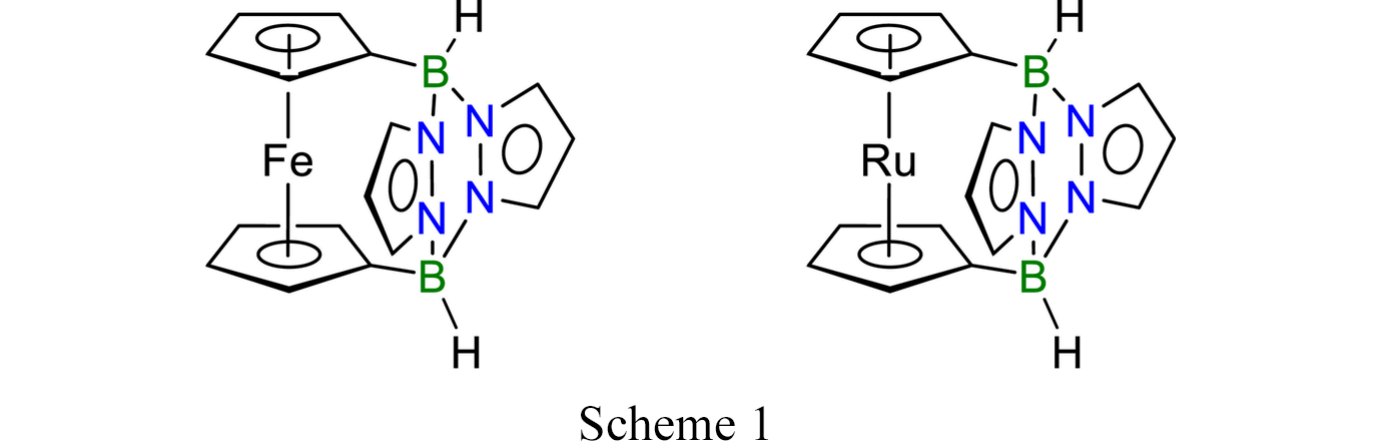


## 2. Experimental

### 2.1. Synthesis

We describe the synthesis of the novel compound Rc(BHpz)_2_. For the synthesis of Fc(BHpz)_2_, see Henkelmann *et al.* (2022 ▸). Ruthenocene was synthesized according to literature procedures (Rickmeier & Ritter, 2018 ▸).

#### 2.1.1. General considerations

All reactions, manipulations and analyses of air- and moisture-sensitive compounds were carried out under an atmosphere of dry argon or nitrogen using Schlenk techniques or in an argon- or nitrogen-filled glovebox. *n*-Hexane was dried over Na metal; Et_2_O and THF were dried over Na/benzophenone. CH_2_Cl_2_ was distilled from CaH_2_. Prior to use, the solvents were degassed by applying three freeze–pump–thaw cycles and stored over molecular sieves (3 Å). C_6_D_6_ was dried over Na metal without benzophenone (2–3 days), degassed as described above and stored over molecular sieves (3 Å). THF-*d*_8_ was dried over Na/benzophenone, distilled and degassed. CD_2_Cl_2_ was distilled from CaH_2_, degassed and stored over molecular sieves (3 Å).

#### 2.1.2. Rc(Bpin)_2_, compound **1**

Ruthenocene (1.50 g, 6.49 mmol, 1.00 eq) was suspended in 20 mL *n*-hexane and *N*,*N*,*N*′,*N*′′,*N*′′-pentamethyldiethylenetriamine (PMDTA; 3.40 mL, ρ = 0.83 g cm^−3^, 16.28 mmol, 2.51 eq) was added. To this mixture *n*-butyl lithium (7.30 mL, 2.21 M, 16.13 mmol, 2.49 eq) was added dropwise at room temperature and stirring was continued for 3 h. The solvent was reduced to 2 mL and the mixture was cooled to −78°C, dissolved in 20 mL THF and 2-isopropoxy-4,4,5,5-tetramethyl-1,3,2-dioxaborolane (*i*PrOBPin; 3.30 mL, ρ = 0.91 g cm^−3^, 16.14 mmol, 2.49 eq) was added in one portion (see Scheme 2). The mixture was allowed to warm to room temperature and stirred overnight. A saturated aqueous solution of NH_4_Cl (30 mL) was added together with 50 mL of CH_2_Cl_2_. The phases were separated, and the aqueous phase was extracted two times with 20 mL of CH_2_Cl_2_. The combined organic layers were washed with 50 mL of distilled water and 50 mL brine. The organic phase was dried with Na_2_SO_4_, filtered, and all volatiles were removed under reduced pressure. After recrystallization with *n*-hexane the product was obtained as pale-yellow crystals. Yield of 1,1′-bis(4,4,5,5-tetramethyl-1,3,2-dioxaborolane)ruthenocene (**1**): 2.04 g (4.22 mmol, 65%).
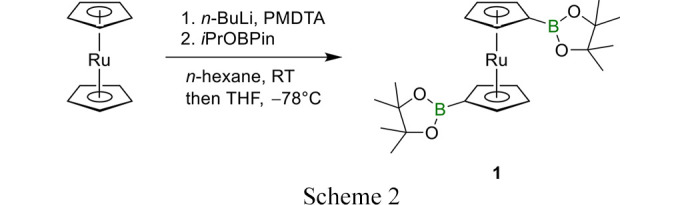


Single crystals of **1** suitable for X-ray diffraction were obtained by recrystallization with *n*-hexane.

#### 2.1.3. Rc(BH_3_Li)_2_, compound **2**

Compound **1** (1.00 g, 2.07 mmol, 1.00 eq) was dissolved in 10 mL Et_2_O and cooled to 0°C in an ice-bath. LiAlH_4_ (0.16 g, 4.21 mmol, 2.04 eq) was suspended in 5 mL Et_2_O and added dropwise over 30 min (Scheme 3). The ice-bath was removed, and the mixture stirred for 1 h at room temperature. After filtration through a G4 porosity frit, the solvent was removed under reduced pressure to obtain a colourless residue. To remove contaminations with Al salts, the solid material was dissolved in a minimal amount of THF (1 mL), and 12-crown-4 (0.70 mL, ρ = 1.09 g cm^−3^, 4.33 mmol, 2.09 eq) was added. The product was precipitated with *n*-hexane (5 mL) and the mother liquid was removed with a syringe. After washing the precipitate three times with 5 mL Et_2_O and drying under reduced pressure, the product was obtained as a colourless solid. Yield of 1,1′-bis(lithium trihydridoborata)­ruthenocene **2**·12-crown-4·(THF)_2_: 0.46 g (0.78 mmol, 38%). 
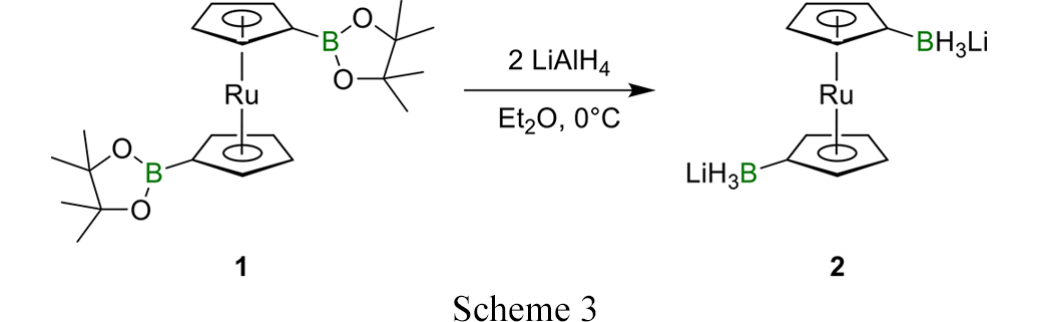


Single crystals of **2**·12-crown-4·(THF)_2_ suitable for X-ray diffraction were obtained after one day by layering a saturated solution of **2**·12-crown-4·(THF)_2_ in THF (0.5 mL) with *n*-hexane (1.5 mL).

#### 2.1.4. Rc(BHpz)_2_, compound **3**

**2**·12-crown-4·(THF)_2_ (0.14 g, 0.24 mmol, 1.00 eq) and 1*H*-pyrazole (0.05 g, 0.73 mmol, 3.06 eq) were dissolved at room temperature in 10 mL THF and stirred for 2 h. Then, the mixture was cooled to −78°C with an isopropanol/dry-ice bath and trimethylchlorosilane (TMSCl; 0.15 mL, ρ = 0.856 g cm^−3^, 1.18 mmol, 4.92 eq) was added dropwise. The cooling-bath was removed and, after warming to room temperature, the mixture was gradually heated to reflux for 5 h (Scheme 4). All volatiles were removed under reduced pressure, and the residue was dissolved in CH_2_Cl_2_, filtered over a plug of silica gel (6 cm) and the solvent was removed under reduced pressure. The solid residue was washed with *n*-hexane, Et_2_O and acetonitrile (5 mL each). After drying under reduced pressure, the product was obtained as a colourless, microcrystalline solid. Yield of 1,1′-(3,6-pyrazabolyl)ruthenocene (**3**): 0.01 g (0.03 mmol, 11%). 
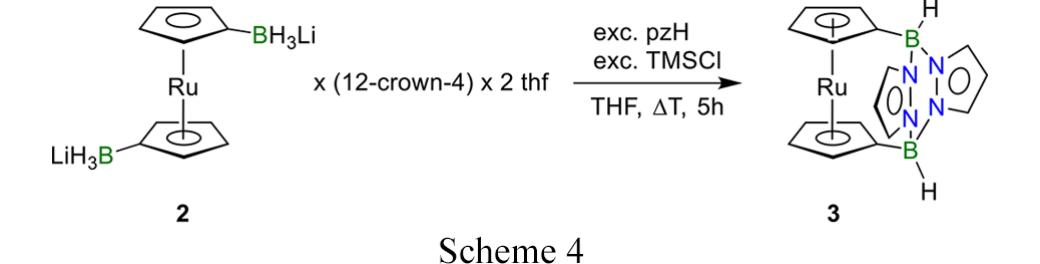


Single crystals of Rc(BHpz)_2_ suitable for X-ray diffraction were obtained by slow evaporation of a saturated solution of Rc(BHpz)_2_ in CH_2_Cl_2_ in an NMR tube in the form of colourless planks.

### 2.2. Single crystal diffraction

Intensity data of two Fc(BHpz)_2_ and one Rc(BHpz)_2_ crystal were collected using Mo*K*α radiation on a STOE IPDS2 diffractometer at 173 K. For improved reciprocal space reconstructions, data of a further Rc(BHpz)_2_ crystal were collected at the P24 beamline at PETRA III, a high brilliance photon source, using 0.5 Å radiation at 100 K. Data of all crystals were converted into the ESPERANTO format and processed with *CrysAlisPro* (Rigaku Oxford Diffraction, 2022 ▸). A correction for absorption and beam inhomogeneity was applied using the multi-scan approach.

For structure refinements the three crystals measured using the IPDS2 diffractometer system were used, because the P24 data set features a more complex diffraction pattern (see below). The structures were solved with *SHELXT* (Sheldrick, 2015*b* ▸) and refined with *SHELXL* (Sheldrick, 2015*a* ▸).

As detailed below, one Fc(BHpz)_2_ crystal and the Rc(BHpz)_2_ crystal were composed of multiple domains, yet the structure was solved and refined using only the major triclinic domain. Twin refinements including all reflections were also attempted, but we found them to be non-advantageous. A brief comparison of the refinements is given at the end of the manuscript. The second Fc(BHpz)_2_ crystal was monoclinic with pseudo-orthorhombic metrics and refined as a twin by pseudo merohedry.

For the triclinic structures, where alternative domains were ignored, residual positive electron densities in difference Fourier maps were interpreted as Fe/Ru atom positions due to alternative stacking arrangements. In total, the Fe/Ru atom was refined as ‘disordered’ over four positions with the sum of the occupancies fixed to 1. The anisotropic atomic displacement parameters (ADPs) of the four positions were constrained to be equal. For the triclinic Fc(BHpz)_2_ structure, also the ligand was refined as positionally disordered about two positions, where the minor position was identified from peaks in the difference electron density map. Note that the Fe/Ru atoms were refined as disordered about more positions than the ligands, which leads to an inconsistency in the reported occupancies. However, given that the alternative Fe/Ru positions are occupied to less than 6%, this appears to be a reasonable choice, preferred over underreporting the total ligand occupancy.

To highlight the layer character of the structures and to better relate them, the atomic coordinates were transformed into unconventional non-reduced settings, such that the layer plane is parallel to (001) [relationship with the reduced cell for the triclinc structures: (**a**, **b**, **c**) = (−**a**_*r*_ + **c**_*r*_, −**b**_*r*_, **a**_*r*_); relationship with the standard cell for the monoclinic structure: (**a**, **b**, **c**) = (−**b**_*r*_, −**a**_*r*_,  −**c**_*r*_)]. Non-H atoms were refined with anharmonic ADPs. H atoms were placed at calculated positions and thereafter refined as riding on the parent atom.

More data collection and refinement details are compiled in Table 1 ▸.

Two-dimensional reciprocal space sections were reconstructed from frame data using the *unwarp* plugin of *CrysAlisPro*. One-dimensional profiles were extracted from *k*-constant sections by summing over multiple pixels for each *l* value.

## 3. Results and discussion

### 3.1. Molecular structure

**Figure 1:**
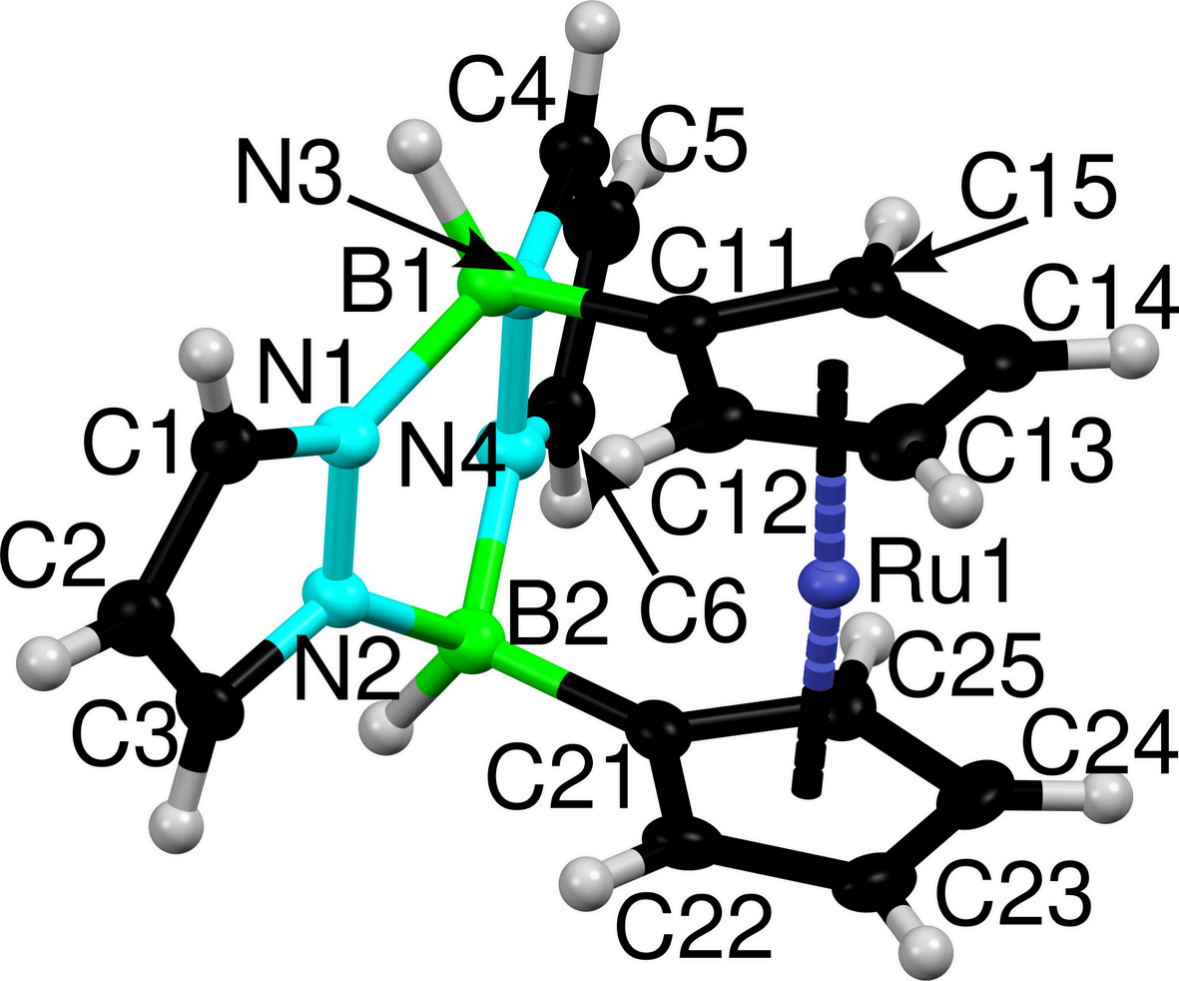
Molecular structure of Rc(BHpz)_2_ with labeling. Atoms are represented by dark blue (Ru), turquoise (N), black (C) and green (B) ellipsoids drawn at the 50% probability level; H atoms drawn as grey spheres of arbitrary radius.

The ligands in Fc(BHpz)_2_ and Rc(BHpz)_2_ feature identical coordination behavior. Exemplarily, Rc(BHpz)_2_ is shown in Fig. 1 ▸, including the atom-numbering scheme. Table 2 ▸ gives a few characteristic geometric parameters for the molecules of the three refined crystal structures. The Fe/Ru—Cp distance is distinctly larger for the ruthenocene compound (*ca* 1.80 versus 1.64 Å), which requires a notably larger angle between the two Cp rings (*ca* 6.9 versus 3.2°).

### 3.2. Polytypes

Fc(BHpz)_2_ and Rc(BHpz)_2_ are polytypic structures, which means that they are built of layers that can be arranged in different ways. Crystals of both compounds are built of isostructural layers, which are arranged according to the same principle. One could speak of members of ‘isostructural’ polytype families, though the term is usually applied to single structures, not families of structures.

**Figure 2:**
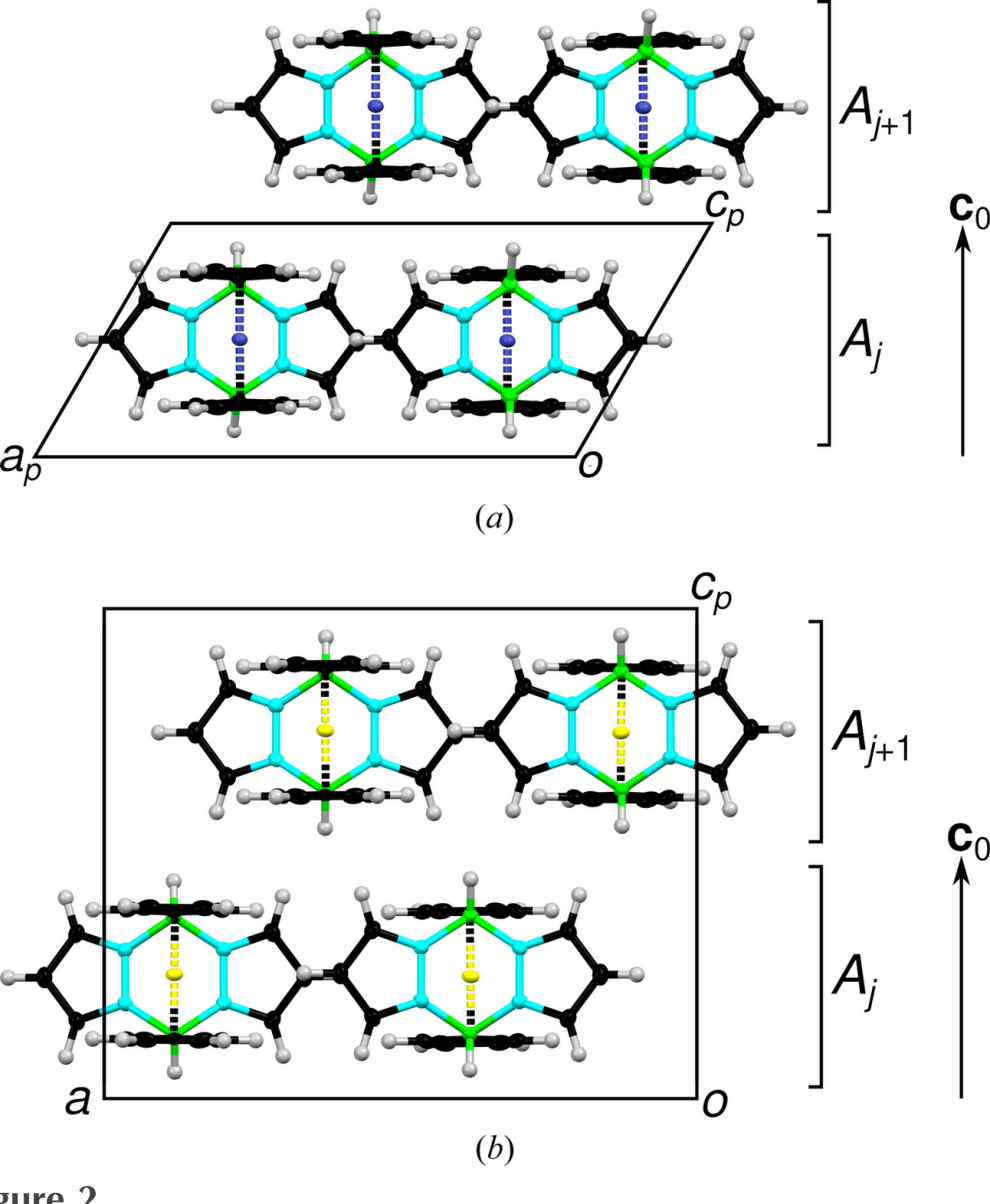
The structures of (*a*) the *P*1 polytype of Rc(BHpz)_2_ and (*b*) of the *P*2_1_/*c*11 polytype of Fc(BHpz)_2_ viewed along [010]. Atom as in Fig. 1 ▸, Fe is yellow. The subscript *p* (for projection) indicates that the axes are, respectively, slightly (*a*_*p*_) and significantly (*c*_*p*_) out of the drawing plane.

The structures of the *P*1 and *P*2_1_/*c*11 polytypes derived from structure refinements are shown in projection along [010] in Fig. 2 ▸. In both cases, one crystallographically unique molecule is located on the general position. The structures are built of layers extending parallel to the (001) plane, which will be called *A*_*j*_, where *j* is a sequential number (see right side of Fig. 2 ▸). The vector perpendicular to the layer planes with the length of one layer width is **c**_0_ as indicated in Fig. 2 ▸. In the *P*1 polytype, all layers are translationally equivalent and two adjacent layers are related by a **c** lattice translation. In *P*2_1_/*c*11, adjacent layers are related by inversions and *n*-glide reflections.

### 3.3. OD interpretation

**Figure 3:**
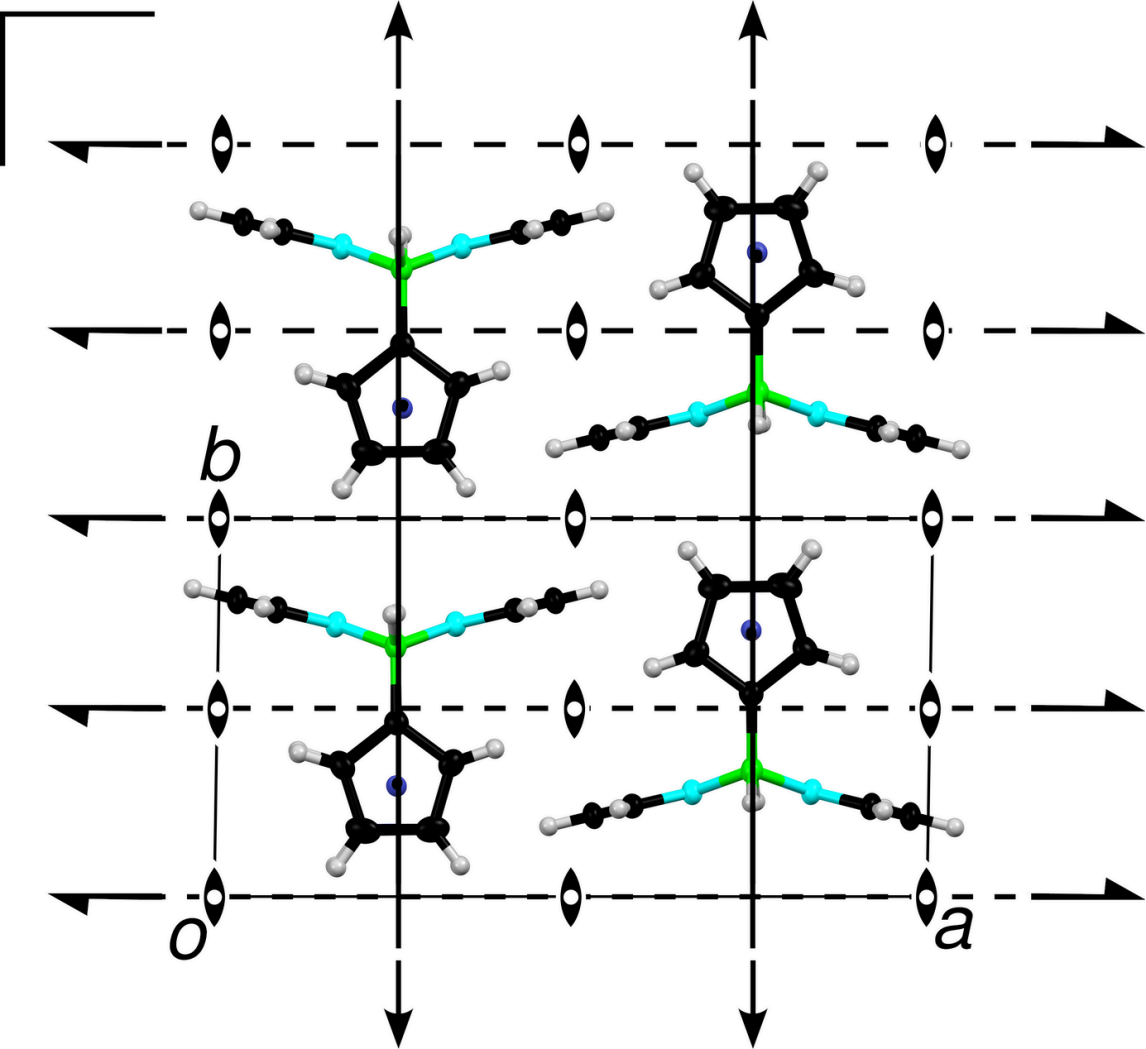
*A*_*j*_ layer in the *P*1 polytype of Rc(BHpz)_2_ projected on the layer plane (001). Atoms as in Fig. 1 ▸. (Pseudo-)symmetry elements are indicated using the common symbols (Hahn & Aroyo, 2016 ▸). The layers in Fc(BHpz)_2_ are virtually identical and therefore not shown.

An OD interpretation is typically based on partial (in the sense of pertaining to only subsets of Euclidean space) pseudo-symmetry operations (POs) mapping layers onto themselves or onto distinct layers. Here, first observe that γ ≈ 90°, which means that the *A*_*j*_ layers possess a pseudo-rectangular lattice (Fig. 3 ▸). Then, note that the Fc(BHpz)_2_/Rc(BHpz)_2_ molecules possess pseudo-*m*2*m* symmetry. When extended to the whole layer all operations of the *m*2*m* group leave the layer invariant up to minor desymmetrization.

Overall, the layers possess *Pma*(*m*) symmetry, where the parentheses mark the direction lacking translation (Dornberger-Schiff, 1959 ▸). The equivalent symbol according to the *International Tables of Crystallography* Vol. E is *pmam* (Kopský & Litvin, 2006 ▸). These symbols are not commonly used in the OD literature, because they cannot represent stacking directions other than [001].

The (pseudo-)symmetry elements of the layers are shown in Fig. 3 ▸. The Fc(BHpz)_2_/Rc(BHpz)_2_ molecules are located around special positions with *m*2*m* symmetry, corresponding to their molecular symmetry.

Henceforth, if not indicated otherwise, we will assume that the *Pma*(*m*) symmetry is perfectly realized. *Pma*(*m*) is a *non-polar* layer group, which means that the interfaces at both sides are symmetrically equivalent (here for example related by the *m*_[001]_ reflection). *A* is the standard designation of non-polar layers, because the *A* letter is itself symmetric by reflection (Dornberger-Schiff, 1982 ▸).

The *A*_*j*_ layers all possess the same translation lattice. Moreover, since they are holohedral (possess the full *mmm* point symmetry of their lattice), they appear only in a single orientation state, which means that all layers in a structure are translationally equivalent. Thus, a polytype is fully determined by the origin of each *L*_*j*_ layer. Let **s**^++^) be the vector that connects the origins of two adjacent layers in the 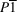 polytype (up to lattice translation). Expressed with respect to the layer lattice basis it is written as 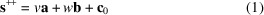
The superscript ++ indicates the sign of the coefficients of **a** and **b**. The three other linear combinations 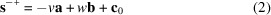

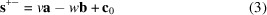

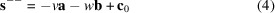
will be used later.

**Figure 4:**
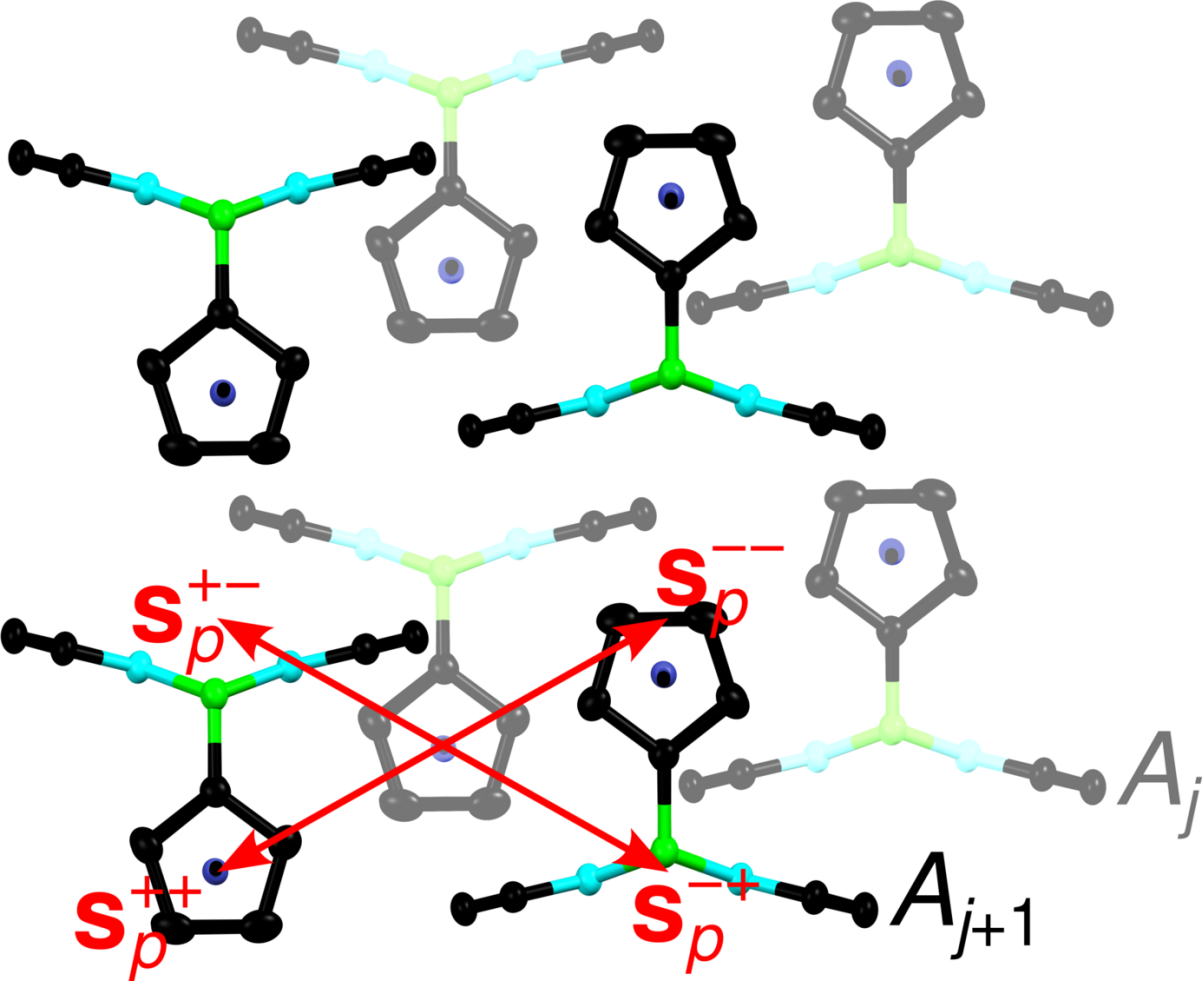
(*A*_*j*_, *A*_*j*+1_) pair of adjacent layers projected on the layer plane (001) with coordinates taken from the Rc(BHpz)_2_ refinement. Atoms as in Fig. 1 ▸, the lower *A*_*j*_ layer has lighter colors. Translation vectors connecting possible layer pairs are given in red. As before, the subscript *p* indicates an out-of-plane component of the vectors.

A pair (*A*_*j*_, *A*_*j*+1_) of adjacent layers whose origins are connected by **s**^++^ is shown in Fig. 4 ▸.

The partial symmetry of OD structures is classified into OD groupoid families, which play the role of space group types in classical crystallography. According to the notation of Dornberger-Schiff & Grell-Niemann (1961 ▸), the partial symmetry of a polytype of the title compounds is a member of the OD groupoid family 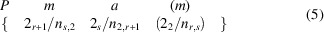 The first line states the layer group, the second line one set of possible operations relating adjacent layers using a generalization of the classical Hermann–Mauguin notation. *r* and *s* are additional metric parameters (Fichtner, 1979*b* ▸) that specify the relative intrinsic translation components of screw rotations and glide reflections relating adjacent layers. The *n*_*r*, *s*_ symbol stands for a glide reflection with intrinsic translation 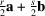. The meaning of the remaining symbols is summarized in Table 3 ▸. Observe that the factors 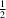 in 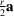 and 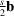 are due to reflections being the improper analogue of a twofold rotation and 2_1_ standing for a screw rotation with the intrinsic translation of half a lattice vector. The parameters (*r*, *s*) are related to the components *v* and *w* [see equation (1)] by *r* = 2*v* and *s* = 2*w*. Here, we find the parameters (*v*, *w*) more convenient and will base the upcoming discussion on them.

### 3.4. Stacking possibilities

Consider a fixed layer *A*_*j*_. POs that fix the layer interfaces (do not invert the layer with respect to the stacking direction) are called λ-τ-POs in the OD literature and form the layer group *Pma*(2) (*pma*2 according to the ITC-E). The subset of operations that map the adjacent layer *A*_*j*+1_ onto itself forms the translation group *P*11(1) (*p*1 according to the ITC-E), because *v* and *w* are neither integer nor half-integer numbers and therefore the *m*_[100]_ and the *a*_[010]_ planes do not overlap.

An operation in *Pma*(2), but not in *P*11(1) fixes the *A*_*j*_ layer but maps *A*_*j*+1_ onto a different position. Thus, it creates additional equivalent pairs of layers given a fixed *A*_*j*_. The *Pma*(2) operations can be grouped into [*Pma*(2):*P*11(1)] = 4 cosets (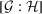 is the *index* of the subgroup 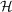 in the supergroup 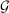), which correspond to four different ways of placing *A*_*j*+1_ given *A*_*j*_ (Ďurovič, 1997 ▸).

Since all layers are translationally equivalent, the simplest way to describe the four ways of placing *A*_*j*+1_ given *A*_*j*_ is the translation vector connecting the origins of *A*_*j*_ to *A*_*j*+1_, which will be called *stacking vectors*. One of these vectors has been given in equation (1). The three others are obtained by applying an operation of the corresponding coset to the translation **s**^++^ (potentially followed by reduction with a layer lattice vector): **s**^+−^, **s**^−+^ and **s**^−−^ [see equations (2)–(4)].

The four stacking vectors are shown in Fig. 4 ▸. Taking the Fe/Ru atom in the *A*_*j*_ layer as starting point, the end points correspond to the four Fe/Ru positions (one main and three ‘phantom atoms’) obtained in the structure refinements.

### 3.5. MDO polytypes and twinning

Given a family of OD polytypes, *i.e.* the set of all polytypes with equivalent pairs of layers, the polytypes of a *maximum degree of order* (MDO) are those that cannot be decomposed into fragments of simpler polytypes (Dornberger-Schiff, 1982 ▸). They play an important role in OD theory, as all other polytypes can be decomposed into fragments of MDO polytypes.

In crystals of the title compounds, all (*A*_*j*_, *A*_*j*+1_) pairs of layers are equivalent (as required by the definition of OD structures), but there are four kinds of (*A*_*j*_, *A*_*j*+1_, *A*_*j*+2_) triples (corresponding to the four different ways of placing *A*_*j*+1_ given *A*_*j*_, see §3.4). These define the four MDO polytypes, which are built of only one kind of these triples:

  MDO_1_: …**s**^++^**s**^++^…, *P*1, **c** = **s**^++^.

  MDO_2_: …**s**^++^**s**^+−^…, *P*2/*c*, **c** = 2**c**_0_ + 2*v***a**.

  MDO_3_: …**s**^++^**s**^−+^…, *P*2_1_/*c*11, **c** = 2**c**_0_ + 2*w***b**.

  MDO_4_: …**s**^++^**s**^−−^…, *P*112_1_/*m*, **c** = 2**c**_0_.

The point group of the OD groupoid family is the group generated by the linear parts of the POs of all polytypes. Here, it is *mmm*. By coset decompositions of the point group of a polytype in *mmm* one obtains the possible orientation states of a polytype (or fragment thereof) in a disordered stacking arrangement. Here we have:

  MDO_1_: 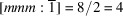,

  MDO_2_: [*mmm*:2/*m* − −] = 8/4 = 2,

  MDO_3_: [*mmm*: − 2/*m* −] = 8/4 = 2,

  MDO_4_: [*mmm*: − − 2/*m*] = 8/4 = 2

possible orientations (2/*m* − − stands for the point group 2/*m* with unique axis [100], *etc*.). Stacking faults in macroscopic domains of MDO polytypes may cause twinning, by switching between the possible orientation states.

### 3.6. Metric parameters

The OD groupoid family given in equation (5) abstracts from metric parameters, just as the 230 space group types abstract from the cell parameters. These parameters are the layer unit-cell dimensions, layer width and the relative origin shifts *v* and *w*. The former correspond to the *a* and *b* parameters given in Table 1 ▸. The fundamental domain of the layer lattice is slightly shrunk in Ru(BHpz)_2_ compared to Fc(BHpz)_2_ (area *ca* 116 versus 119 Å^2^).

The layer widths calculated from the unit-cell parameters are 
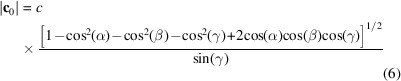
or, simplified by idealizing γ to 90°: 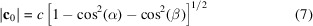
for the MDO_1_ (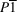) polytypes and 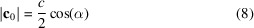
for the MDO_3_ (*P*2_1_/*c*11) polytype. They are listed in the first line of Table 4 ▸ for the three refined structures. As expected, the layers in Rc(BHpz)_2_ are thicker (*ca* 6.39 versus 6.16 Å) owing to the larger Ru–Cp distance.

The relative origin shifts can be calculated for the MDO_1_ polytypes according to 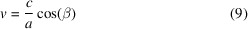

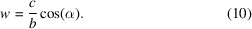
For MDO_3_, only *w* can be derived from the unit-cell parameters according to 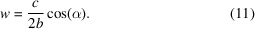
The parameter *v* is derived from the atomic coordinates: averaging the *y* coordinates (excluding H) atoms and subtracting 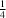 gives the relative distance of the layer’s pseudo-reflection plane from the *c* glide plane of the crystal. Twice this distance is *w*. *v* and *w* are listed at the bottom of Table 4 ▸. Observe that they show very little variation across polytypes (*i.e.* there is little desymmetrization). The crucial points for interpreting the diffraction patterns in the next section is that they can be approximated as *v*, *w* ≈ 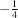.

### 3.7. Diffraction pattern

To show the complexity that can arise from families of OD structures such as the title compound, we will discuss the diffraction patterns of four different crystals, which were all obtained by slow evaporation from CH_2_Cl_2_. Two Fc(BHpz)_2_ crystals will be called Fc1 (source of the *P*1 refinement of Fc(BHpz)_2_) and Fc2 (source of the *P*2_1_/*c*11 refinement of Fc(BHpz)_2_). Data for both were collected using the IPDS2 diffractometer system. The data of Fc1 was used to publish the original structure of Fc(BHpz)_2_ (Henkelmann *et al.*, 2022 ▸). The two Rc(BHpz)_2_ crystals will be called Rc1 and Rc2. Both were extracted from the same synthesis batch. Data of Rc1 was collected using the IPDS2 diffractometer [source of the *P*1 refinement of Rc(BHpz)_2_], of Rc2 at the P24 beamline (no refinement performed, owing to broad additional peaks, hampering intensity evaluation).

The diffraction patterns will all be indexed with respect to the reciprocal basis 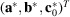, the dual of the real space basis (**a**, **b**, **c**_0_). Note that by convention reciprocal bases are given as columns, hence the superscript *T*.

**Figure 5:**
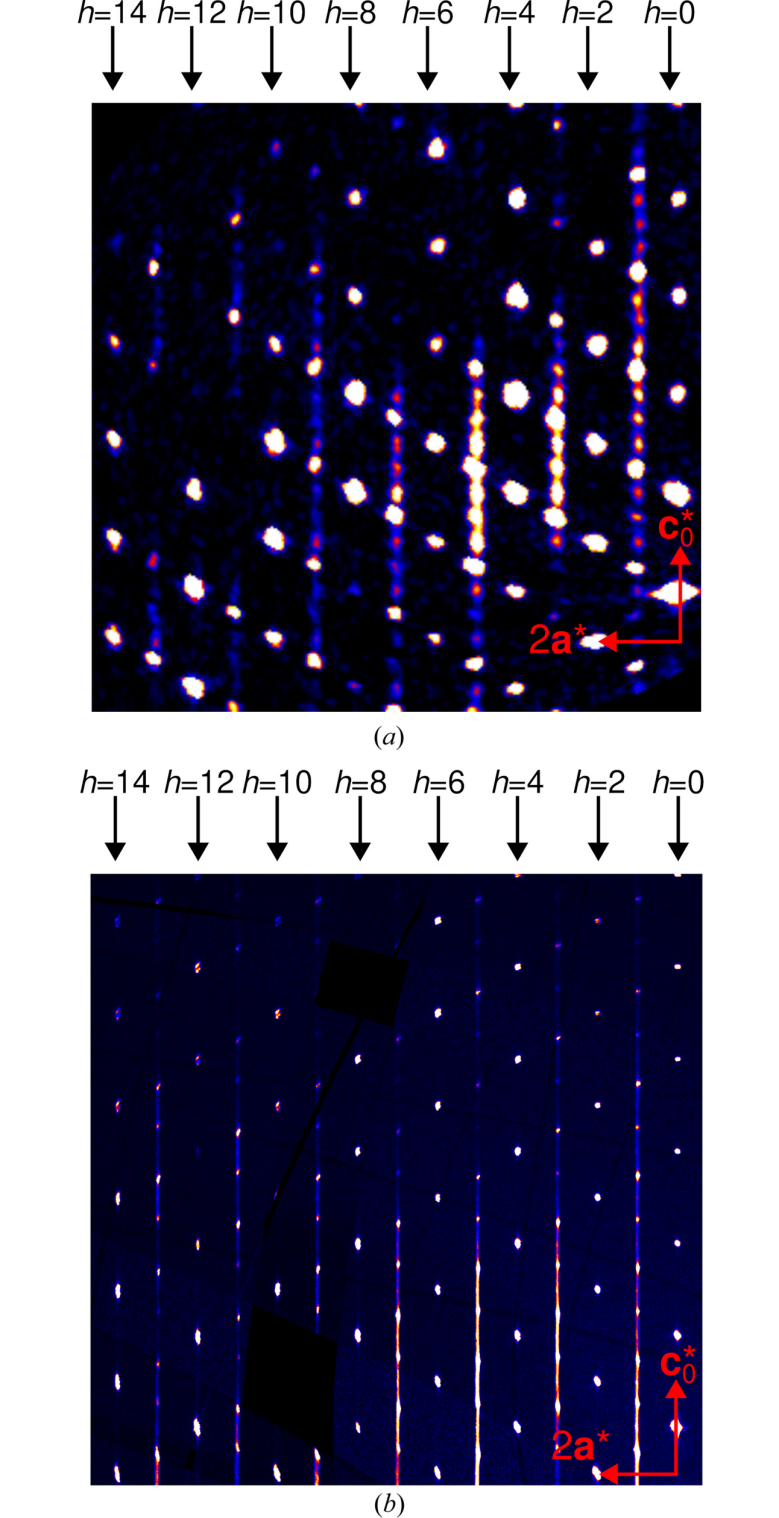
Reconstructions of the *k* = 2 plane of reciprocal space of crystals (*a*) Fc1 and (*b*) Rc2 (measured at P24), showing the family reflections on rods *h*, *k* even. Black arrows mark *h*, *k* even rows with family reflections.

A feature common to all investigated crystals is that for *h* and *k* even, diffraction intensities are only observed for (*h* + *k*)/2 + *l* = 2*n*, 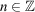, as shown exemplarily for Fc1 and Rc2 in Fig. 5 ▸. These reflections correspond to the *family structure* [*Immm* symmetry, 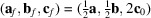], an equal overlay of all possible stacking arrangements, when assuming perfect translational equivalence of all layers and idealizing the metric parameters to *v*, *w*= −¼. They are therefore called *family reflections*. All stacking arrangements, disordered or not, produce only sharp (Bragg) reflections on rods *h* and *k* even, as can be derived by a standard argument [see e.g. Ferraris *et al.* (2008 ▸)]. Very faint streaks are due to deviation from the idealized model [called *desymmetrization*, see Ďurovič (1979 ▸)]. Their faintness however shows that the idealization is legitimate (Fig. 5 ▸).

### 3.8. Macroscopic domains

On rods *h* or *k* odd, the diffraction patterns contain discrete peaks and/or diffuse components, whereby we consider as discrete those peaks whose profile is not broader than those of the family reflection when scaled to the same intensity. There, one can assume that the profile is dominated by experimental artifacts (radiation: spectrum, finite coherence length, divergence; crystal: finite size and mosaicity, desymmetrization, *etc*.).

These sharp peaks are due to macroscopic ( > the coherence length of the radiation) domains of particular periodic polytypes and are called *characteristic reflections* in the OD literature. From the **c** vectors of the MDO polytypes given above, one can infer the reciprocal bases of the four MDO polytypes (assuming ideal *v*, *w*= −¼):

  MDO_1_: 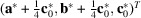


  MDO_2_: 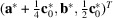


  MDO_3_: 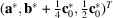


  MDO_4_: 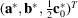


Observe that the basis of MDO_1_ does only index family reflections on rods *h* and *k* even. For MDO_2, 3, 4_ though, one would expect additional reflection between the family reflections, because c*= 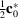. These non-existing reflections are called *non-spacegroup* or *non-crystallographic* systematic absences and are a characteristic phenomenon of polytypic structures of translationally equivalent layers.

As noted above, for MDO_1_, there are four possible orientations, which we will designate according to one representative operation with respect to the reference **c** = −¼**a** − ¼**b** + **c**_0_ domain as the 1, 2_[100]_, 2_[010]_ and 2_[001]_ domains. 2_[001]_ here stands for a rotation about **c**_0_, *i.e.* a direction perpendicular to the layer plane. The corresponding reciprocal bases are:

  MDO_1_ (1): 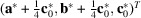


  MDO_1_ (2_[100]_): 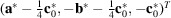


  MDO_1_ (2_[010]_): 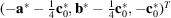


  MDO_1_ (2_[001]_): 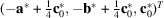


These bases index reflections at distinct positions on rods *h* and/or *k* odd. In contrast, MDO_2, 3, 4_ are monoclinic, yet their lattices are orthorhombic (assuming ideal 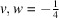), and thus the twin domains produce reflections at the same positions [twinning by pseudo-merohedry (Grimmer & Nespolo, 2006 ▸)]. The expected positions for all MDO polytypes, and their twin domains in the case of MDO_1_, is compiled in Table 5 ▸.

**Figure 6:**
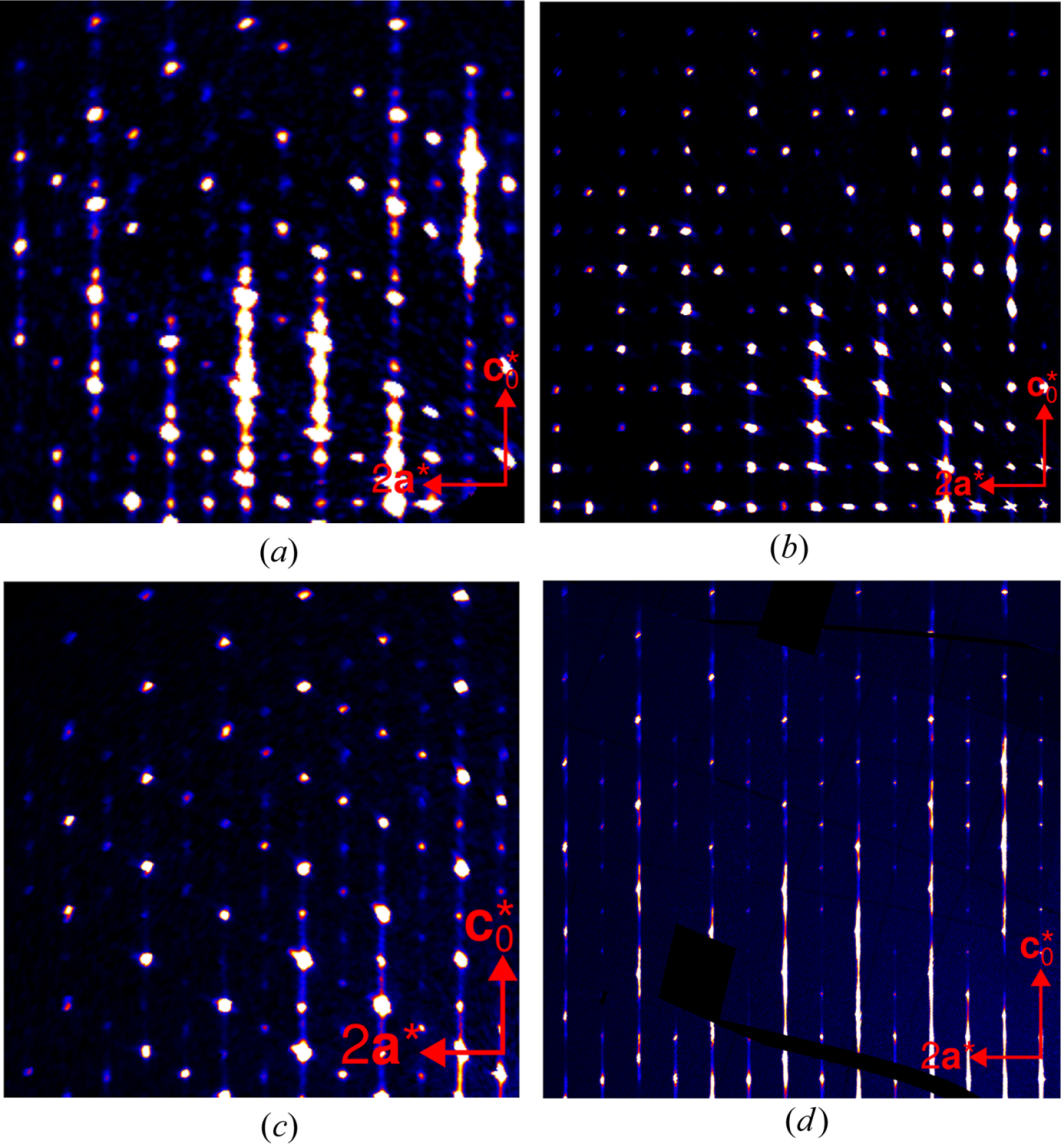
Reconstructions of the *k* = 1 planes of reciprocal space of crystals (*a*) Fc1, (*b*) Fc2, (*c*) Rc1 and (*d*) Rc2.

**Figure 7:**
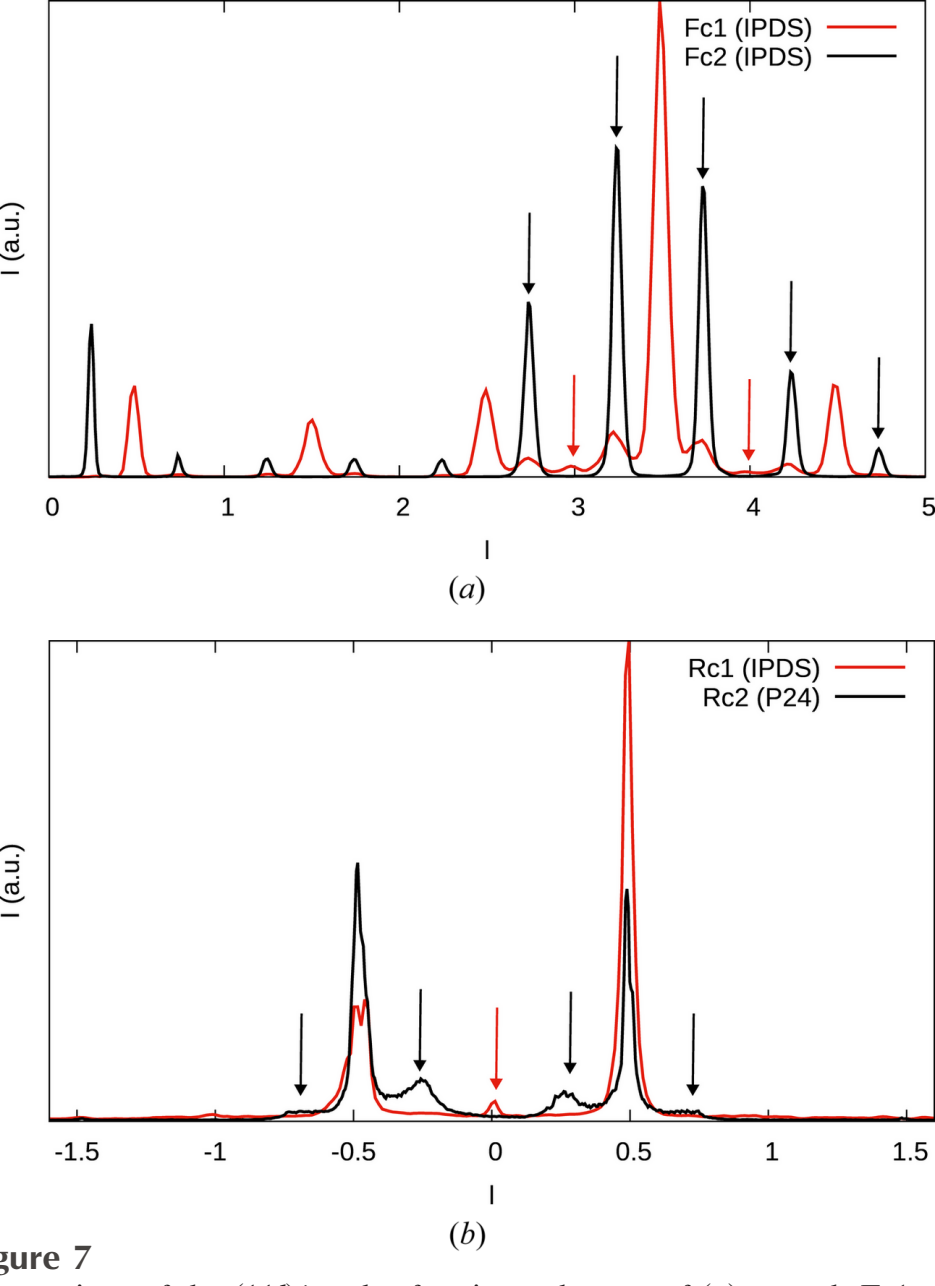
Comparison of the (11*l*)* rods of reciprocal space of (*a*) crystals Fc1 and Fc2 and (*b*) crystals Rc1 and Rc2. Black arrows indicate position of MDO_3_ reflections, red arrows the positions of the 2_[100]_, 2_[001]_ orientations of MDO_1_.

No sharp peaks were observed for any crystal on *h* even, *k* odd rods with half-integer *l* (see Fig. 6 ▸). Thus, according to Table 5 ▸, long-range ordered domains of MDO_2_ and MDO_4_ can be excluded. Crystals Fc1, Rc1 and Rc2 featured sharp reflections on rods *h* and *k* odd with integer and half-integer *l* (see Fig. 7 ▸), meaning that there are macroscopic MDO_1_ domains (observe that MDO_4_ was excluded). Crystal Fc1 and Fc2 featured sharp reflections on rods *h* and *k* odd with odd fourths of *l* (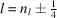) [black arrows in Fig. 7 ▸(*a*)], indicating macroscopic MDO_3_ domains (MDO_2_ having been excluded). Rc2 likewise features peaks at these positions [black arrows in Fig. 7 ▸(*b*)], however they are distinctly more diffuse than the family reflections and therefore not due to macroscopic MDO_3_ domains. In summary, the macroscopic domains are:

  Fc1: MDO_1_ and MDO_3_

  Fc2: MDO_3_

  Rc1: MDO_1_

  Rc2: MDO_1_

Since Fc1 is built of *different* polytypes, it is classified as an *allotwin* (Nespolo *et al.*, 1999 ▸).

For the crystals containing MDO_1_, namely Fc1, Rc1 and Rc2, the fractions of the four twin domains are remarkably unequally distributed. To quantify the fractions, reflection intensities were evaluated by integrating using the orthorhombic basis (**a**, **b**, 4**c**_0_). For Rc1 and Rc2, which do not contain sharp MDO_3_ reflections, an *oI* centering was applied which indexes the reflections of all four MDO_1_ orientations (and spurious reflections on the rods of family reflections). For Fc1 a primitive setting was used, to also integrate the MDO_3_-only reflections. The average intensities of each reflection class (normed to an arbitrary intensity of 1000 for the family reflections) are compiled in Table 6 ▸.

It is striking that in all three crystals, there is one dominant domain (which was used as the reference with orientation 1). The second most dominant domain is obtained by twofold rotation about **c**_0_. The two others (twofold rotation about **a** and **b**) are much less pronounced (see red arrows in Fig. 7 ▸). In particular in crystal Rc2 (data collected at the P24 beamline and therefore of high quality) there are no reflections of the 2_[100]_,2_[010]_ domains and the crystal therefore contains only two ordered MDO_1_ domains [see red arrows in Fig. 7 ▸(*b*)]. The small integrated intensity of these ‘reflections’ (line 5 in Table 6 ▸) is an artifact due to one-dimensional diffuse scattering. In Fc1 and Rc1, on the other hand, all four orientations exist. Crystal Fc1 features MDO_3_ reflections, which are much less pronounced, though, than the MDO_1_ reflections [last line in Table 6 ▸, black arrows in Fig. 7 ▸(*a*)].

Crystal Fc2 featured reflections only of MDO_3_ [black arrows in Fig. 7 ▸(*b*)], which can appear in two orientation (see above). To determine the twin volume ratio, the structure was refined as a twin by pseudo-merohedry. Including the alternative twin domain improved the residuals slightly (*R*_obs_ from 0.036 to 0.032). However, the fraction of the minor domain refined to only barely one percent [0.0110(2)]. We suspect that the improvement of the residuals is not due to an actual second domain but to erroneous intensity evaluations caused by weak diffuse scattering. This phenomenon has been called the *Ďurovič effect* (Nespolo & Ferraris, 2001 ▸).

In summary, the crystals under investigation are composed of the following long-range ordered domains:

  Fc1: MDO_1_ (1, 2_[100]_, 2_[010]_, 2_[001]_), MDO_3_

  Fc2: MDO_3_ (only one domain)

  Rc1: MDO_1_ (1, 2_[100]_, 2_[010]_, 2_[001]_)

  Rc2: MDO_1_ (1, 2_[001]_)

in strongly varying fractions, which suggests a very small stacking fault density. For a large number of stacking faults, the law of large numbers predicts pairs of twin domains with approximately equal volume ratios.

### 3.9. Diffuse scattering

**Figure 8:**
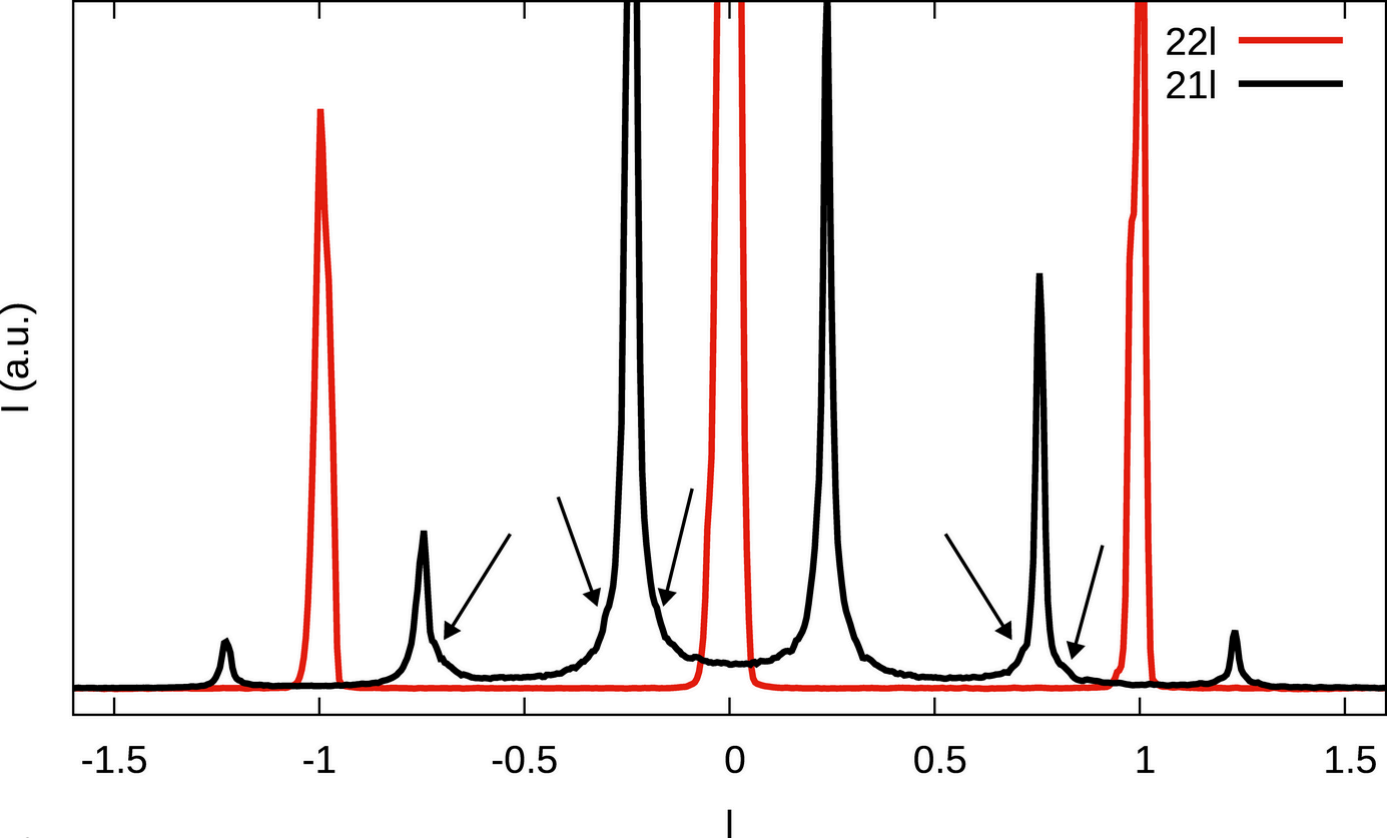
Comparison of the family reflections on the (22*l*)* rod (red) and characteristic reflections of the MDO_1_ polytypes on the (12*l*)* rod (black) of crystal Rc2. Black arrows indicate a broadening at the base, which we attribute to a disordered domain.

All four crystals under investigation feature one-dimensional diffuse scattering along 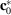, on rods *h* or *k* odd, which is in contradiction to the low stacking fault density. We therefore suppose that the crystals are built of ordered and disordered domains. This can for example be caused by slow transformation of disordered to ordered domains, as we have described in Stöger *et al.* (2021 ▸). In fact, while the central parts of the MDO_1_ characteristic reflections in Rc2 are generally as sharp as the family reflections, at the base one observes a distinct broadening, which we interpret as the contributions of a more disordered domain (black arrows in Fig. 8 ▸).

Since the diffraction patterns are dominated by the sharp reflections, we will only discuss the diffuse components qualitatively. The diffuse scattering is distinctly more pronounced in the case of the Rc(BHpz)_2_ crystals than the Fc(BHpz)_2_ crystals (see Fig 6 ▸) and we will therefore focus the discussion on the former.

We will designate as a *simple* disordered domain a domain that is built of two kinds of MDO fragments (for example a mix of MDO_1_ and MDO_2_ fragments). Such a simple model can be regarded as a simplified OD family with a lower-symmetry family structure. Accordingly, as can be shown with structure factor considerations [as for example those given in Ferraris *et al.* (2008 ▸) or Stöger *et al.* (2021 ▸)], under the idealizations given above streaks would be observed at most on two kinds of rods, as compiled in Table 7 ▸.

**Figure 9:**
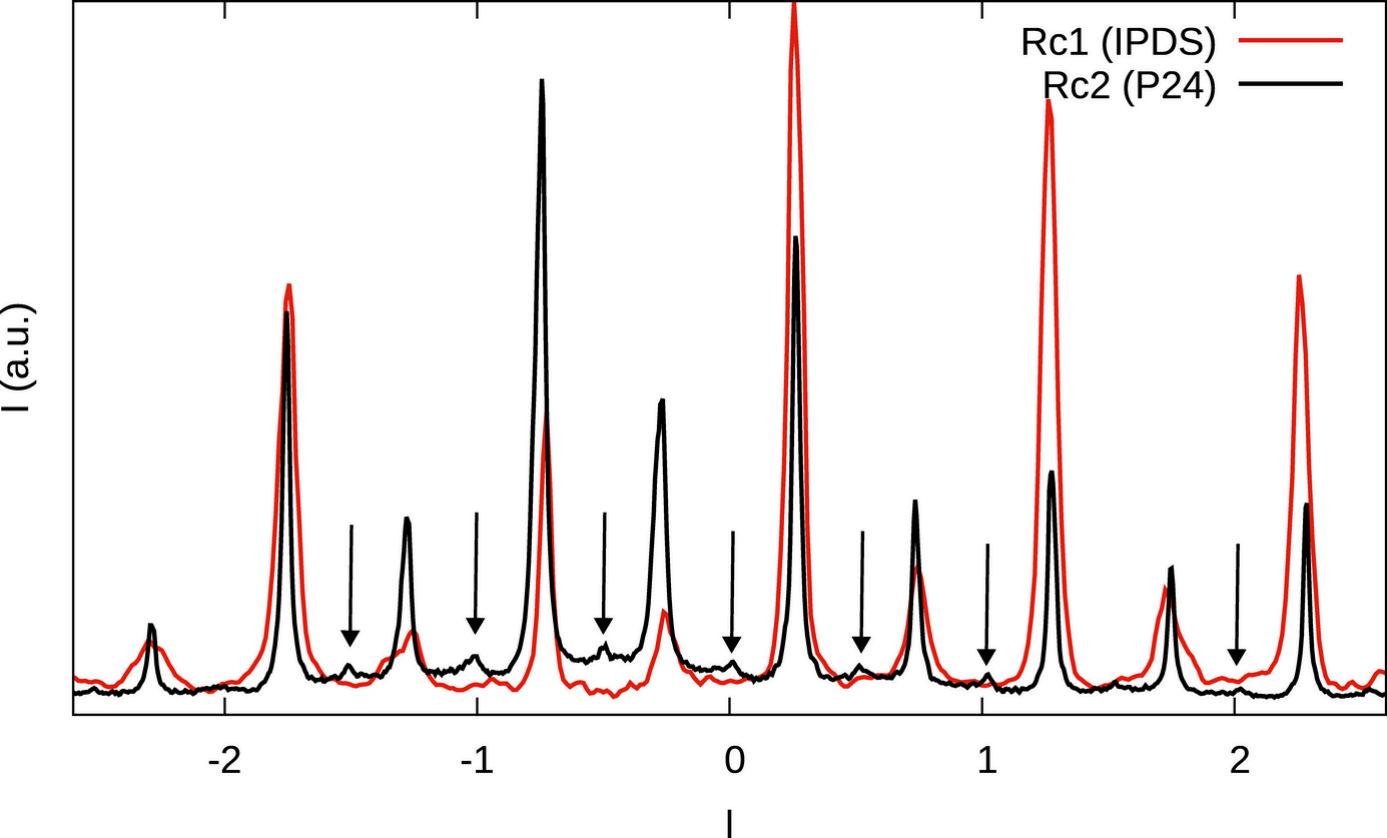
Comparison of the (12*l*)* rods of reciprocal space of crystals Rc1 and Rc2. Black arrows indicate peaks observed in crystal Rc2 but not crystal Rc1.

The Bragg peaks of the models with MDO_1_ fragments would be located under the corresponding peaks of the ordered fragments (rows 1–3 in Table 7 ▸) and therefore cannot be seen. The sharp peaks of non-MDO_1_ models (rows 4–6 in Table 7 ▸) would be visible. No such peaks are observed for crystal Rc1 ruling out such models. There are though tiny peaks at *l* = *n*/2 on rods *h* odd, *k* even in crystal Rc2 (black arrows in Fig. 9 ▸). This might indicate disordered MDO_3_/MDO_4_ domains, though the peaks are too weak to judge whether they are sharp or diffuse.

In any case, for both Rc(BHpz)_2_ crystals, diffuse streaks are observed on *all* rods with either *h* and/or *k* odd, which means that either the crystals possess simple disordered domains of different kind (*e.g.* MDO_1_/MDO_3_*and* MDO_3_/MDO_4_) or the disordered domains are more complex (*i.e.* an MDO_1_ fragment can be followed by any other fragment). Twinning cannot explain the diffuse scattering on all rods, as the twin operations don’t affect the parity of *h* or *k*.

### 3.10. Additional diffraction features in crystal Rc2

As noted above, crystal Rc2 features additional broad peaks at *l* ≈ (2*n* + 1)/4 on rods *h* and *k* odd that are not observed in crystal Rc1 [black arrows in Fig. 7 ▸(*b*)]. The peaks are consistent with MDO_3_ fragments. Given that the additional peaks on rods *h* odd, *k* even (black arrows in Fig. 9 ▸) are very weak, it is not currently possible to determine the exact nature of these domains. Overall, we conclude that the diffuse scattering of the Rc(BHpz)_2_ crystals is due to a complex combination of ordered and disordered domains, which are expressed to a different extent in different crystals.

### 3.11. Comparison of refinements

Despite the samples being twinned, the structural refinements based on the intensity data of crystals Fc1 and Rc1 were performed using only reflections of the major domain. In this section, we compare this simple refinement of Rc1 to a ‘proper’ twin refinement. It has to be noted that determination of twin volume ratios and stacking fault frequencies by classical refinements is treacherous, as we have for example shown for KAgCO_3_ (Hans *et al.*, 2015 ▸). The crystals under investigation are particularly problematic, since they feature both ordered and disordered parts and therefore intensity evaluation of individual reflections will be affected by the diffuse scattering.

As noted in the *Experimental*[Sec sec2], when ignoring the twinning, the alternative stacking arrangements are observed as ‘phantom atoms’. These may be ‘real’ in the sense that there is coherent diffraction between different domains, they may be due to unaccounted for twinning (addition of |*F*|^2^, not *F*) or they may be an artifact owing to erroneous intensity evaluations (the *Ďurovič effect* mentioned above). These contributions usually cannot be separated.

For comparison we attempted refinements as a twin of the two major orientation states (related by 2_[001]_, see above) and even with all four orientation states. The twin volume ratios and the intensity of the ‘phantom atoms’ are compared in Table 8 ▸. In twin refinements, the occupancies of the ‘phantom atoms’ decreased, but if omitted, they generally reappeared in difference Fourier syntheses. Only the minor position in the four-domain twin refinement could not be located any longer and was omitted.

Overall, we found the refinements of similar quality. Apparently, the interatomic distances are adequately determined by the intensities of the family reflection. The residuals are nominally better when using only the main domain, owing to improved intensities. It is unclear whether including the minor domains is advantageous, since the phantom atoms do not fully disappear anyway. It has been suggested to place different reflection classes on different scale factors, though this likewise is just cosmetics by adding new parameters: it is not given that the Ďurovič effect is linear with intensity. As noted above, the twin volume ratios and occupancies are unreliable in any case.

## 4. Conclusion and outlook

The title compounds Fc(BHpz)_2_ and Rc(BHpz)_2_ prove that polytypes can possess a fascinatingly complex crystallization behavior. Small changes in chemistry, such as substitution of Fe by Ru may lead to different crystallization behavior. Yet, even from the same crystallization attempt very different crystals are extracted, hinting towards a chaotic system. To fully understand the nature of the diffuse scattering, additional experiments will be necessary. In particular, we expect insights from temporal changes in the diffraction pattern due to a rearrangement of a kinetic structure to the thermodynamic one. But also different crystallization conditions such as temperature and solvents may shed more light on the disorder model.

The OD formalism allows a unified description of the polytype family and provides a convincing rationale for the ambiguity in the stacking sequence.

## 5. Related literature

The following reference is cited in the supporting information: Fulmer *et al.* (2010 ▸).

## Supplementary Material

Crystal structure: contains datablock(s) Fc(BHpz)2triclinic, Fc(BHpz)2monoclinic, Rc(BHpz)2. DOI: 10.1107/S2052520625009758/ra5154sup1.cif

Structure factors: contains datablock(s) FcBHpz2triclinic. DOI: 10.1107/S2052520625009758/ra5154FcBHpz2triclinicsup2.hkl

Structure factors: contains datablock(s) FcBHpz2monoclinic. DOI: 10.1107/S2052520625009758/ra5154FcBHpz2monoclinicsup3.hkl

Structure factors: contains datablock(s) RcBHpz2. DOI: 10.1107/S2052520625009758/ra5154RcBHpz2sup4.hkl

Crystal structures of the intermediate products. DOI: 10.1107/S2052520625009758/ra5154sup5.txt

NMR data. DOI: 10.1107/S2052520625009758/ra5154sup6.pdf

CCDC references: 2500070, 2500071, 2500072

## Figures and Tables

**Table 1:** Experimental details

	Fc(BHpz)_2_	Fc(BHpz)_2_	Rc(BHpz)_2_
Crystal data			
Chemical formula	C_16_H_16_B_2_FeN_4_	C_16_H_16_B_2_FeN_4_	C_16_H_16_B_2_N_4_Ru
*M*_r_	341.80	341.80	387.02
Crystal system, space group	Triclinic, *P*1	Monoclinic, *P*2_1_/*c*11	Triclinic, *P*1
Temperature (K)	173	173	173
*a*, *b*, *c* (Å)	14.9044 (11), 7.9751 (6), 7.5243 (8)	14.9315 (2), 7.9951 (1), 12.9916 (3)	14.7902 (7), 7.8656 (5), 7.6804 (4)
α, β, γ (°)	106.184 (8), 120.367 (10), 89.339 (6)	108.513 (2), 90, 90	105.700 (5), 119.274 (4), 89.096 (4)
*V* (Å^3^)	732.00 (13)	1470.66 (5)	743.04 (8)
*Z*, *Z*′	2, 1	4, 1	2, 1
Radiation type	Mo *K*α	Mo *K*α	Mo *K*α
μ (mm^−1^)	1.03	1.03	1.06
Crystal shape	Block	Prism	Plank
Colour	Light brown	Orange-brown	Colourless
Crystal size (mm)	0.29 × 0.22 × 0.14	0.38 × 0.22 × 0.20	0.24 × 0.06 × 0.02

Data collection			
Diffractometer	STOE IPDS2	STOE IPDS2	STOE IPDS2
Absorption correction	Multi-scan	Multi-scan	Multi-scan
*T*_min_, *T*_max_	0.754, 0.869	0.696, 0.821	0.696, 0.821
No. of measured, independent and observed [*I* > 2σ(*I*)] reflections	6102, 2809, 2533	33085, 4585, 4186	7594, 3504, 3263
*R*_int_	0.026	0.038	0.030
(sin θ/λ)_max_ (Å^−1^)	0.617	0.716	0.659

Refinement			
*R*[*F*^2^ > 2σ(*F*^2^)], *wR*(*F*^2^), *S*	0.036, 0.090, 1.10	0.032, 0.090, 1.12	0.035, 0.092, 1.09
No. of reflections	2809	4585	3504
No. of parameters	288	209	221
No. of restraints	21	0	1
H-atom treatment	H-atom parameters constrained	H-atom parameters constrained	H-atom parameters constrained
Δρ_max_, Δρ_min_ (e Å^−3^)	0.37, −0.35	0.56, −0.34	1.25, −0.72

Computer programs: *SHELXL2014/7* (Sheldrick, 2015*a* ▸).

**Table 2:** Geometric parameters of Fc(BHpz)_2_ and Rc(BHpz)_2_ derived from the three structure refinements Angles between and distances to the Cp rings are calculated with respect to the least-squares planes.

	Fc(BHpz)_2_ (*P*1)	Fc(BHpz)_2_ (*P*2_1_/*c*11)	Rc(BHpz)_2_ (*P*1)
Angle between Cp rings (°)	3.15 (17)	3.22 (4)	6.86 (18)
Distance between Cp centroid and Fe/Ru atom (Å)	1.6432 (12), 1.6427 (12)	1.6430 (6), 1.6454 (6)	1.8004 (12), 1.8020 (12)
Distance of B from Cp plane (Å)	0.069 (4), 0.073 (4)	0.068 (2), 0.070 (2)	0.112 (6), 0.112 (5)

**Table 3:** Meaning of the symbols in the second line of equation (5)

Symbol	Direction	Intrinsic translation
2_*r*+1_	[100]	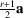
*n*_*s*, 2_	[100]	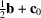
2_*s*_	[010]	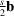
*n*_2, *r*+1_	[010]	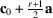
2_2_	[001]	**c**_0_
*n*_*r*, *s*_	[001]	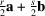

**Table 4:** Metric parameters of the three polytypes under investigation: layer width |**c**_0_| and relative origin shifts *v* and *w* (see text)

	Fc(BHpz)_2_ (*P*1)	Fc(BHpz)_2_ (*P*2_1_/*c*11)	Rc(BHpz)_2_ (*P*1)
|**c**_0_| (Å)	6.159	6.160	6.388
*v*	−0.255	−0.255	−0.254
*w*	−0.263	−0.258	−0.264

**Table 5:** Reflection conditions for the four MDO polytypes Polytypes are given by their number; for MDO_1_ the orientations are given in parentheses. 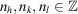.

*k*	*l*	*h* = 4*n*_*h*_	*h* = 4*n*_*h*_ + 1	*h* = 4*n*_*h*_ + 2	*h* = 4*n*_*h*_ + 3
4*n*_*k*_	*n*_*l*_	All (family)	3, 4	–	3, 4
	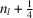	–	1 (1, 2_[010]_), 2	–	1 (2_[100]_, 2_[001]_), 2
	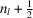	–	3, 4	All (family)	3, 4
	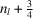	–	1 (2_[100]_, 2_[001]_), 2	–	1 (1, 2_[010]_), 2

4*n*_*k*_ + 1	*n*_*l*_	2, 4	1 (2_[100]_, 2_[010]_), 4	2, 4	1 (1, 2_[001]_), 4
	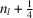	1 (1, 2_[100]_), 3	2,3	1 (2_[010]_, 2_[001]_), 3	2, 3
	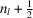	2, 4	1 (1, 2_[100]_), 4	2, 4	1 (2_[100]_, 2_[010]_), 4
	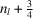	1 (2_[010]_, 2_[001]_), 3	2, 3	1 (1, 2_[100]_), 3	2, 3

4*n*_*k*_ + 2	*n*_*l*_	–	3, 4	All (family)	3, 4
	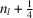	–	1 (2_[100]_, 2_[001]_), 2	–	1 (1, 2_[010]_), 2
	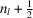	All (family)	3, 4	–	3, 4
	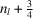	–	1 (1, 2_[010]_), 2	–	1 (2_[100]_, 2_[001]_), 2

4*n*_*k*_ + 3	*n*_*l*_	2, 4	1 (1, 2_[001]_), 4	2, 4	1 (2_[100]_, 2_[010]_), 4
	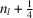	1 (2_[010]_, 2_[001]_), 3	2, 3	1 (1, 2_[100]_), 3	2, 3
	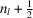	2, 4	1 (2_[100]_, 2_[010]_), 4	2, 4	1 (1, 2_[001]_), 4
	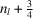	1 (1, 2_[100]_), 3	2, 3	1 (2_[010]_, 2_[001]_), 3	2, 3

**Table 6:** Reflection classes of the four orientations of the MDO_1_ polytype in the crystals Fc1, Rc1 and Rc2 (potentially including MDO_3_ intensity) and the MDO_3_ only reflections in crystal Fc1 Intensities are averaged over all reflections of the class, normalized to the average of the family reflections. Pure MDO_3_ reflections of Rc1 and Rc2 were not integrated (use of *oI* instead of *oP* cell), since they were either absent or strongly diffuse.

Class	Fc1	Rc1	Rc2
Family	1000	1000	1000
1 / 2_[100]_ / (MDO_3_)	552	493	461
1 / 2_[010]_	465	446	390
1 / 2_[001]_	876	835	555
2_[100]_ / 2_[010]_	28	34	5
2_[100]_ / 2_[001]_	57	78	95
2_[010]_ / 2_[001]_ / (MDO_3_)	93	72	140
MDO_3_	85	–	–

**Table 7:** Positions of Bragg peaks and diffuse scattering on rods *h* or *k* odd in models involving two kinds of MDO fragments Positions of Bragg peaks are given with *n* standing for an integer.

Model	*h* odd, *k* even	*h* even, *k* odd	*h* and *k* odd
MDO_1_/MDO_2_	Bragg at *l* = (*h* + *k*)/4 + *n*	Diffuse	Diffuse
MDO_1_/MDO_3_	Diffuse	Bragg at *l* = (*h* + *k*)/4 + 1	Diffuse
MDO_1_/MDO_4_	Diffuse	Diffuse	Bragg at *l* = (*h* + *k*)/4 + *n*
MDO_2_/MDO_3_	Diffuse	Diffuse	Bragg at *l* = (2*n* + 1)/4
MDO_2_/MDO_4_	Diffuse	Bragg at *l* = *n*/2	Diffuse
MDO_3_/MDO_4_	Bragg at *l* = *n*/2	Diffuse	Diffuse

**Table 8:** Comparison of structure refinements with one, two or four twin domains The twin domains and occupancies of the Ru atoms are given by the operation with respect to the major domain.

	One domain	Two domains	Four domains
Twin volume ratio			
1	1	82.36 (10)	77.0 (2)
2_[100]_	–	–	1.32 (15)
2_[010]_	–	–	4.45 (16)
2_[001]_	–	17.64 (10)	17.3 (2)
Ru occupancy ratio			
1	92.08 (10)	94.75 (17)	95.4 (2)
2_[100]_	0.35 (8)	0.62 (11)	–
2_[010]_	1.55 (8)	1.68 (1)	1.36 (15)
2_[001]_	6.02 (7)	2.96 (12)	3.27 (18)
*R*_obs_	0.0345	0.0496	0.0750
*wR*(*F*^2^)_obs_	0.0917	0.1267	0.2047
*R*_all_	0.0377	0.0618	0.0983
*wR*(*F*^2^)_all_	0.0894	0.1286	0.2083
No. of parameters	221	222	220

## References

[bb1] Cliffe, M. J., Wan, W., Zou, X., Chater, P. A., Kleppe, A. K., Tucker, M. G. H., Wilhelm, P., Funnell, N., Coudert, F.-X. & Goodwin, A. L. (2014). *Nat. Commun.* **5**, 4176. 10.1038/ncomms5176 PMC4730551 24946837

[bb2] Dornberger-Schiff, K. (1959). *Acta Cryst.* **12**, 173–173.

[bb3] Dornberger-Schiff, K. (1982). *Acta Cryst.* A**38**, 483–491.

[bb4] Dornberger-Schiff, K. & Grell-Niemann, H. (1961). *Acta Cryst.* **14**, 167–177.

[bb5] Ďurovič, S. (1979). *Cryst. Res. Technol.* **14**, 1047–1053.

[bb6] Ďurovič, S. (1997). *EMU Notes Mineral.* **1**, 3–28.

[bb7] Ferraris, G., Makovicky, E. & Merlino, S. (2008). *Crystallography of Modular Materials*. IUCr Monograph on Crystallography 15. Oxford University Press.

[bb8] Fichtner, K. (1979*a*). *Cryst. Res. Technol.* **14**, 1073–1078.

[bb9] Fichtner, K. (1979*b*). *Cryst. Res. Technol.* **14**, 1453–1461.

[bb10] Fulmer, G. R., Miller, A. J. M., Sherden, N. H., Gottlieb, H. E., Nudelman, A., Stoltz, B. M., Bercaw, J. E. & Goldberg, K. I. (2010). *Organometallics* **29**, 2176–2179.

[bb11] Grimmer, H. & Nespolo, M. (2006). *Z. Kristallogr.* **221**, 28–50.

[bb12] Hahn, T. & Aroyo, M. I. (2016). In *International Tables For Crystallography*, Vol. A, *Space-group symmetry*, ch. 2.1.2, pp. 144–148. Chester: IUCr.

[bb13] Hans, P., Stöger, B., Weil, M. & Zobetz, E. (2015). *Acta Cryst.* B**71**, 194–202. 10.1107/S2052520615004138 25827372

[bb14] Henkelmann, M., Omlor, A., Bolte, M., Schünemann, V., Lerner, H. W., Noga, J., Hrobárik, P. & Wagner, M. (2022). *Chem. Sci.* **13**, 1608–1617. 10.1039/d1sc06404e PMC8826627 35282635

[bb15] Hope, H. & Karlin, K. D. (1994). *Prog. Inorg. Chem.* **41**, 1–19.

[bb16] Kopský, V. & Litvin, D. B. (2006). Editors. *International Tables For Crystallography*, Vol. E*, Subperiodic groups*. Chester: IUCr.

[bb17] Meekel, E. G., Schmidt, E. M., Cameron, L. J. D., Dharma, A. J., Windsor, H. G., Duyker, S. A., Minelli, A., Pope, T., Lepore, G. O., Slater, B., Kepert, C. J. & Goodwin, A. L. (2023). *Science* **379**, 357–361. 10.1126/science.ade5239 36701437

[bb18] Nespolo, M. & Ferraris, G. (2001). *Eur. J. Mineral.* **13**, 1035–1045.

[bb19] Nespolo, M., Kogure, T. & Ferraris, G. (1999). *Z. Kristallogr.* **214**, 5–8.

[bb20] Peresypkina, E., Stöger, B., Dinauer, S. & Virovets, A. V. (2022). *Cryst. Growth Des.* **22**, 3870–3874.

[bb21] Rickmeier, J. & Ritter, T. (2018). *Angew. Chem. Int. Ed.* **57**, 14207–14211. 10.1002/anie.201807983 30187598

[bb22] Rigaku Oxford Diffraction, (2022). *CrysAlisPro Software System*, version 1.171.42.69a. Rigaku Oxford Diffraction, Yarnton, England.

[bb23] Roth, N. & Goodwin, A. L. (2023). *Nat. Commun.* **14**, 4328. 10.1038/s41467-023-40063-w PMC10356831 37468516

[bb24] Sheldrick, G. M. (2015*a*). *Acta Cryst.* C**71**, 3–8.

[bb25] Sheldrick, G. M. (2015*b*). *Acta Cryst.* A**71**, 3–8.

[bb26] Stöger, B., Krüger, H. & Weil, M. (2021). *Acta Cryst.* B**77**, 605–623.

