# Supplementary material for: The complex stacking disorder of Fe- and Ru-based 1,1′-(3,6-pyrazabolyl)metallocenes
Source: Acta Crystallogr B Struct Sci Cryst Eng Mater. 2026 Feb 1;82(Pt 1):22–33. doi: 10.1107/S2052520625009758 (PMC12869525; doi:10.1107/S2052520625009758)
Supplement: Supplementary file 6 [file b-82-00022-sup6.pdf]

The complex stacking disorder of Fe- and Ru-based 1,1′-(3,6-pyrazabolyl)metallocenes — Supporting Information

Berthold Stöger Alexandr Virovets Mischa Wenisch

NMR Data

If not stated otherwise, NMR spectra were recorded at 298 K using the following spectrometer: *Bruker Avance*™ *III* 500 HD. Chemical shift values are referenced to (residual) solvent signals ($^{1}H/^{13}C\{^{1}H\}$; C_6D_6: $\delta = 7.16/128.06$ ppm; CD_2Cl_2: $\delta = 5.32/53.84$ ppm); THF-d_8: $\delta = 3.58/67.21$ ppm (Fulmer *et al.*, 2010), external $BF_3{\cdot}OEt_2$ or external LiCl (1 M in D_2O) (0.00 ppm). Abbreviations: *s* = singlet, *d* = doublet, *t* = triplet, *q* = quadruplet, *vtr* = virtual triplet, *m* = multiplet, *br* = broad, *n.r.* = not resolved. Resonances of carbon atoms attached to boron atoms were typically broadened and sometimes only observed in ^{1}H-^{13}C-HMBC NMR experiments due to the quadrupolar relaxation of the $^{11/10}B$ nucleus. Resonance assignments were aided by ^{1}H-^{1}H-COSY, ^{1}H-^{13}C-HSQC, ^{1}H-^{13}C-HMBC, ^{1}H-^{13}C-H2BC and ^{1}H-^{1}H-NOESY NMR experiments.

Rc(Bpin)$_2$, compound 1

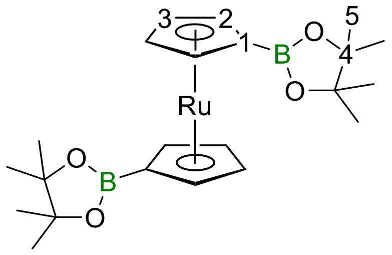

1

^{1}H NMR (500.2 MHz, C_6D_6): $\delta = 5.07$ (*vtr*, $^{3}J_{H,H} = 1.7$ Hz, 4H, H-3), 4.69 (*vtr*, $^{3}J_{H,H} = 1.7$ Hz, 4H, H-2), 1.13 (*s*, 24H, H-5). $^{13}C\{^{1}H\}$ NMR (125.8 MHz, C_6D_6): $\delta = 83.1$ (C-4), 76.2 (C-3), 74.4 (C-2), 64.9 (*br*, C-1) 24.9 (C-5). ^{11}B NMR (160.5 MHz, C_6D_6): $\delta = 32.5$ (*br s*).

Rc(BH$_3$Li)$_2$, compound 2

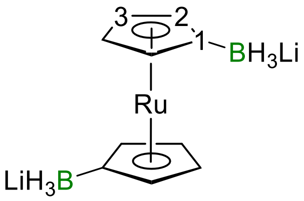

2

^{1}H NMR (500.2 MHz, THF-d_8): δ = 4.22 (*vtr*, $^3J_{H,H}$ = 1.5 Hz, 4H, H-3), 4.01 (*n.r.*, 4H, H-2), 3.71 (*s*, 16H, crown ether-H), 3.62 (*m*, 8H, THF-H-2), 1.77 (*m*, 8H, THF-H-3), 0.78 (*q*, $^1J_{H,B}$ = 81 Hz, 6H, B-H). ^{1}H{^{11}B} NMR (500.2 MHz, THF-d_8): δ = 4.22 (*n.r.*, 4H, H-3), 4.01 (*n.r.*, 4H, H-2), 3.71 (*s*, 16H, crown ether-H), 3.62 (*m*, 8H, THF-H-2), 1.77 (*m*, 8H, THF-H-3), 0.78 (*s*, 6H, B-H). ^{13}C{^{1}H} NMR (125.8 MHz, THF-d_8): δ = 89.5 (*q*, $^1J_{C,B}$ = 51 Hz C-1), 77.5 (C-2), 70.1 (crown ether-C), 69.7 (*br s*, C-3) 68.4 (THF-C-1), 26.5 (*s*, THF-C-2). ^{11}B NMR (160.5 MHz, THF-d_8): $\delta = -32.3$ (*q*, $^1J_{B,H}$ = 81 Hz). ^{11}B{^{1}H} NMR (160.5 MHz, THF-d_8): $\delta = -32.3$ (*s*). ^{7}Li NMR (194.4 MHz, THF-d_8): $\delta = 0.00$ (*s*).

Rc(BHpz)$_2$, compound 3

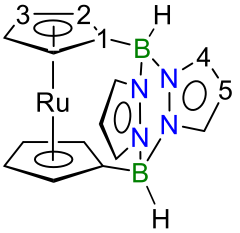

3

^{1}H NMR (500.2 MHz, CD_2Cl_2): δ = 7.75 (*d*, $^3J_{H,H}$ = 2.3 Hz, 4H, H-4), 6.44 (*t*, $^3J_{H,H}$ = 2.3 Hz, 2H, H-5), 4.47 (*br*, 2H, B-H), 4.44 (*vtr*, $^3J_{H,H}$ = 1.6 Hz, 4H, H-3), 3.74 (*vtr*, $^3J_{H,H}$ = 1.6 Hz, 4H, H-2). ^{1}H{^{11}B} NMR(500.2 MHz, CD_2Cl_2): δ = 7.75 (*d*, $^3J_{H,H}$ = 2.3 Hz, 4H, H-4), 6.44 (*t*, $^3J_{H,H}$ = 2.3 Hz, 2H, H-5), 4.47 (*s*, 2H, B-H), 4.44 (*vtr*, $^3J_{H,H}$ = 1.6 Hz, 4H, H-3), 3.74 (*vtr*, $^3J_{H,H}$ = 1.6 Hz, 4H, H-2). ^{13}C{^{1}H} NMR (125.8 MHz, CD_2Cl_2): δ = 136.3 (C-4), 106.1 (C-5), 84.2 (*br*, C-1), 72.4 (C-2), 72.4 (C-3). ^{11}B NMR (160.5 MHz, CD_2Cl_2): $\delta = -4.4$ (d, $^1J_{B,H}$ = 103 Hz). ^{11}B{^{1}H} NMR (160.5 MHz, CD_2Cl_2): $\delta = -4.4$ (*s*).

References

Fulmer, G. R., Miller, A. J. M., Sherden, N. H., Gottlieb, H. E., Nudelman, A., Stoltz, B. M., Bercaw, J. E. and Goldberg, K. I. (2010). *Organometallics*, **29**, 2176–2179.
